# Linking personality traits and reproductive success in common marmoset (*Callithrix jacchus*)

**DOI:** 10.1038/s41598-022-16339-4

**Published:** 2022-08-03

**Authors:** Michaela Masilkova, David Boukal, Hayley Ash, Hannah M. Buchanan-Smith, Martina Konečná

**Affiliations:** 1 Department of Zoology, Faculty of Science, University of South Bohemia, České Budějovice, Czech Republic; 2 Department of Game Management and Wildlife Biology, Faculty of Forestry and Wood Sciences, Czech University of Life Sciences, Prague, Czech Republic; 3 Department of Ecosystem Biology, Faculty of Science, University of South Bohemia, České Budějovice, Czech Republic; 4 Czech Academy of Sciences, Biology Centre, Institute of Entomology, České Budějovice, Czech Republic; 5 Wisconsin National Primate Research Center, University of Wisconsin, Madison, USA; 6 Psychology, Faculty of Natural Sciences, University of Stirling, Stirling, Scotland, UK; 7 Scottish Primate Research Group, Stirling, Scotland, UK

**Keywords:** Ecology, Evolution, Psychology, Zoology

## Abstract

Animal personality can affect individual fitness and population growth. Personality traits of either parent or parents’ combination may facilitate reproduction and offspring survival across species. However, previous studies focused mainly on the role of only one sex, and the link between personality and fitness has not been confirmed in primates. We examined this link in both sexes of captive common marmosets (*Callithrix jacchus*), a cooperatively breeding primate with extensive paternal care. We studied the effects of five personality traits of the parents (Agreeableness, Assertiveness, Conscientiousness, Inquisitiveness, and Patience), including their absolute and directional differences within pairs, on key components of reproductive performance. We expected pairs with more similar personality scores to have higher reproductive success as found in other species with long-term pairs and biparental care, but found no evidence for this hypothesis. Instead, we detected strong effects of female traits on inter-birth intervals, which were shorter in more agreeable females, and fecundity rates, which were higher in more inquisitive females. Male traits appeared to have only a limited effect on reproductive success of the pair. Our study demonstrates that various aspects of animal personality underpin reproductive performance in captive common marmosets and provides novel insights into the possible ultimate causes of personality in cooperatively breeding species.

## Introduction

Stable individual differences in behaviour, termed ‘animal personalities’, have a genetic basis and fitness implications, and as such are subjected to evolutionary processes^1,2^. Across species, personality has been linked to various aspects of reproductive performance, from the number of sperm to the number of successfully weaned infants and infants’ condition^3–6^. In theory, selection should favour personality types with higher reproductive success (reviewed in Smith and Blumstein^2^), and lead to a gradual erosion of population-level variation in personality over evolutionary timescales. Yet, this outcome is not observed, with different personality types existing within the population. Although research has progressed over the past years, the mechanisms maintaining different personality types in animal populations have not been fully explained^1,7^.

Various theories have been advanced to explain the presence of personality types^7–12^. Fluctuating selection and life history trade-offs are the two mechanisms that have so far received the most empirical support^13–17^. For instance, less aggressive and exploratory females of wild boars raise more juveniles to independence than aggressive and exploratory ones but only in years with high food availability^6^. Less docile males of bighorn sheep reproduce earlier in their life but have shorter life expectancy compared to docile and bold males that reproduce later but survive longer^18^. Studies on reproductive implications of personality traits, however, often focus only on one parent, typically mothers^6,19^. Personality traits of the other parent or personality combination of both parents, rather than their individual values, can also affect their fitness and thus contribute to the maintenance of personality types within populations^11^. On one hand, assortative mating of parents with matching personalities can be beneficial and mediated through enhanced behavioural compatibility, fertilisation success, mate fidelity, and effective parental coordination in monogamous species with biparental infant care^5,20,21^. On the other hand, disassortative mating of parents with dissimilar personalities can be adaptive in promiscuous species without biparental care if certain personality trait values facilitate copulation or when parents benefit from producing phenotypically variable offspring or offspring with intermediate trait values^21–23^. The association between the type of personality matching, mating system and level of paternal care, however, does not hold absolutely^23,24^ and might be affected by the species, energetic costs of infant care and environmental fluctuations^14^. Furthermore, these studies focused on a few selected personality traits (e.g. exploration^20^) or reproductive variables (e.g. mating success^23^) and did not consider other potentially relevant personality (e.g. sociability) and reproductive traits (e.g. speed of reproduction). As a result, our understanding of the links between personality traits of the parents and individual fitness is limited.

Although studies examining the fitness consequences of parental personality traits in mammals are on the increase^20,22,23,25^, this relationship remains surprisingly underexplored in primates, a diverse order with various social and reproductive strategies. Among other things, the involvement of primate males in infant care varies from none to extensive paternal care^26^. We are aware of a single study on rhesus macaques, which found no effect of mother’s personality traits on the duration of inter-birth interval or infant survival^19^.

To fill these gaps, we systematically examined the links between several traits of both parents’ personalities and reproductive success for the first time in a cooperatively breeding primate. The common marmoset (*Callithrix jacchus*) is a New World callitrichid naturally adapted to give birth to twin offspring that are taken care of by all group members including the father^27^. It is used widely in biomedical research, often studied in captivity^28^, and laboratory colonies usually have detailed breeding records of their marmosets covering long periods of time. While breeding and social organisations are flexible in the wild^29^, marmosets in captivity are bred most successfully as monogamous pairs, housed in family groups^30^. Callitrichids are characterised by early sexual maturity, multiple ova per cycle, multiple infants per litter, postpartum oestrus, short inter-birth intervals, and no menopause^27^. As a result, callitrichid females have the highest lifetime reproductive potential among non-human primates^31^. However, these reproductive variables can vary considerably between pairs^31,32^, which has been attributed to diverse factors including length of inter-birth interval^33^, litter size^34^, infant body mass^35^, maternal body mass^36^, age^31^, number of previous litters^37^, experience with rearing younger siblings^38^, group size^39^, and housing conditions^35^. However, none of these factors, either alone or in combination, can fully explain the observed variation in reproductive success in callitrichids^27,31^. Personality traits of breeding partners could therefore contribute to the variation in reproductive success among callitrichid pairs.

Our study sample were common marmosets (N = 21 pairs) living in a captive colony with a long breeding history. We considered five validated traits (Table 1) of common marmoset personality structure^40–42^ derived from rating individuals on an adjective-based personality questionnaire^43^. The reproductive variables covering different aspects of reproductive output, from the pace of reproduction to offspring survival and fecundity rate, were acquired from breeding records and demographic data of the colony (n = 560 infants). We tested the direct and distinct effects of each breeding partner personality trait and partners’ trait combination (see *explanatory variables* in Table 2) on number of reproductive variables (see *response variables* in Table 2), while controlling for other relevant variables in a series of mixed effect models. Based on previous findings in species with long-term mate relationships and biparental care^5,20^, we predicted that pairs with more similar personality scores in some traits will have higher reproductive success due to increased parental coordination and behavioural compatibility^44^. As some studies have shown^45,46^, personality of single partner rather than the partners’ personality combination might drive the reproductive success. Therefore, we also tested the distinct effects of male and female personality traits.

**Table 1:** Common marmoset’s personality structure derived from questionnaire ratings based on Koski et al.^40^.

Dimensions	Abbreviation	Items
Conscientiousness^a^	*co*	− thoughtless, − bullying, − clumsy, − eccentric, − reckless, − disorganised, − imitative, − erratic, − jealous, − aggressive, − irritable, − impulsive, − excitable, − unperceptive, − socially playful, − depressed, − stingy, − playful, − assertive
Agreeableness	*ag*	+ friendly, + equable, + affectionate, + permissive, + gentle, + sociable, + popular, + helpful, + predictable, + unemotional, + protective
Assertiveness^a^	*as*	− cautious, − dependent, + dominant, + independent, + confident, − timid, − submissive, − fearful, − tense, − anxious, − vulnerable, + selective, − sympathetic
Patience	*pa*	− distractible, − quitting, + intelligent, + inventive, + sensitive, + persistent, + patient
Inquisitiveness^a^	*in*	− lazy, + exploratory, + inquisitive, + active, + opportunistic, − solitary, + alert

The resulting personality structure comprises five dimensions (personality traits) characterised by a list of adjectives (items). Positive and negative loadings of items are indicated by + and −, respectively. Each individual then acquires a personality score describing variation between individuals on each dimension, e.g. from less (low score) to more (high score) agreeable individuals.

^a^Loadings reversed to facilitate the interpretation, see^40^.

**Table 2:** Explanatory and response variables included in the models examining the links between personality traits and variables of reproductive success.

Variable name	Symbol	Notes
**Explanatory variables**		
Male trait value	*t*_M_	Computed as unit-weighted *z*-score across all males
Female trait value	*t*_F_	Computed as unit-weighted *z*-score across all females
Mean trait value	*t*	Within pair, *t* = (*t*_*m*_ + *t*_*f*_)/2
Similarity index	|∆*t*|	|∆*t*| =|*t*_*m*_* − t*_*f*_|; low value ~ similar scores, high value ~ dissimilar scores within pair
Signed similarity index	∆*t*	∆*t* = *t*_*m*_* − t*_*f*_; positive value ~ higher score in male, negative value ~ higher score in female
Litter number	*N*	Sequential number of given litter (measure of reproductive history)
Litter size at birth	*B*	Including stillborn offspring
Pair duration	*D*	Duration (in years)
Supplementary feeding	*F*	Binary explanatory variable
**Response variables**		
Length of short inter-birth interval	*L*_IBI(short)_	Only inter-birth intervals shorter than 166 days; measured in days
Length of long inter-birth interval	*L*_IBI(long)_	Only inter-birth intervals longer than 165 days; measured in days
Probability of long inter-birth interval	*P*_long_	Interval longer than 165 days
Litter size at birth	*B*	Number of offspring, including stillborn ones
Probability of infant survival	*s*	Only live-born infants considered; until 3 months of age
Fecundity rate	*R*_1_	Total fecundity per year (total litter size, including stillborn offspring)
Fecundity rate	*R*_2_	Total number of weaned offspring surviving until 3 months of age per year

*t* stands for the actual traits: Conscientiousness (*co*), Agreeableness (*ag*), Assertiveness (*as*), Patience (*pa*), Inquisitiveness (*in*).

## Results

### Inter-birth intervals

Multiple competing models were identified as plausible (all models with ∆AICc ≤ 6, for details see “Materials and methods”) for inter-birth intervals (further in text as IBI) durations *L*_IBI(short)_ and *L*_IBI(long)_ (Table S1 in “Supplementary online materials”). Nevertheless, the most parsimonious models suggested that the length of short IBIs *L*_IBI(short)_ decreased significantly with higher female Agreeableness *ag*_F_ and increased with each subsequent litter *N* in the pair’s reproductive history (*n* = 183; LMM; Table 3, Fig. S1A,B). The length of long IBIs *L*_IBI(long)_ tended to increase with mean Agreeableness *ag*, similarity index of Agreeableness |*∆ag*| (i.e. shorter *L*_IBI(long)_ in partners with similar scores) and each subsequent litter *N* (*n* = 30; LMM; Table 3, Fig. S1C–E).

**Table 3:** Summary of the most parsimonious model for each of the five components of reproductive success.

Predictors	Probability of long IBI *P*_long_		Duration of short IBI *L*_IBI(short)_		Duration of long IBI *L*_IBI(long)_		Litter size *B*		Offspring survival probability *s*	
	Log-odds	df	Estimates	df	Estimates	df	Log-odds	df	Log-odds	df
(intercept)	− 0.63 (− 1.26 to − 0.00)	209	151.8 (150.6–152.9)	48.37	181.7 (104.0–259.4)	19.29	–	–	2.99 (1.91–4.08)	520
*ag*_F_	− **0.77** **(**− **1.27 to **− **0.26)**	209	− **1.31** **(**− **2.47 to **− **0.16)**	19.19	–	–	–	–	–	–
*ag*	–	–	–	–	22.0 (− 20.4 to 64.5)	15.4	–	–	–	–
|∆*ag*|	–	–	–	–	31.6 (− 6.0 to 69.3)	15.44	–	–	0.17 (− 0.02 to 0.35)	520
*co*_M_	–	–	–	–	–	–	− **0.76** **(**− **1.42 to **− **0.10)**	–	–	–
log_10_ (litter number *N*)	− **2.23** **(**− **3.27 to **− **1.19)**	209	**2.73** **(1.71–3.75)**	175.2	37.0 (− 12.2 to 86.2)	15.78	**1.14** **(0.27–2.01)**	–	NA	
Litter number *N*	NA		NA		NA		NA		0.03 (− 0.02 to 0.07)	520
Litter size *B*	NA		NA		NA		NA		− **0.81** **(**− **1.17 to **− **0.45)**	520
Supplementary feeding *F* [yes]	NA		NA		NA		NA		0.03 (− 0.41 to 0.47)	520
**Random effects**										
Residual variance *σ*^2^	1.64		3.81		1273.4		3.29		3.29	
Random effect variance *τ*_0,ID_	0		3.33		5484.9		1.51		0	
Intra-class coefficient ICC	–		0.47		0.81		0.32		–	
Number of groups (pairs) *N* _ID_	21		21		18		21		21	
Observations (*n*)	213		183		30		214		526	
Marginal R^2^/Conditional R^2^	0.386/–		0.195/0.571		0.199/0.849		0.099/0.383		0.089/–	

Parameter estimates given as mean with 95% CI in the parentheses. Parameters significantly different from zero (95% CI does not overlap zero) highlighted in bold. Degrees of freedom (df) for the LMMs approximated by the Kenward-Roger method. *ag* = Agreeableness, *co* = Conscientiousness; _F_ = female, _M_ = male; dash (–) = parameter not retained in the ‘top model’ set; NA = parameter not included as explanatory for the given response.

**Figure 1:**
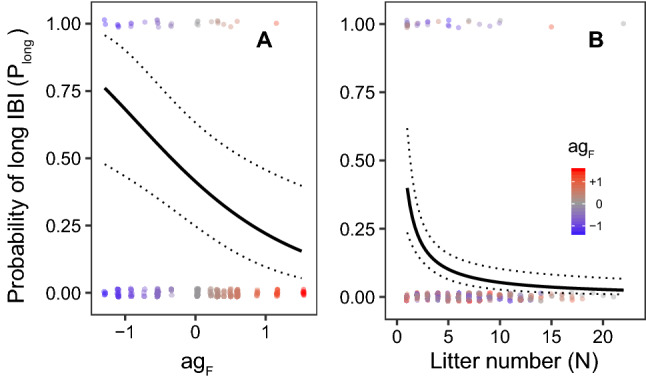
Effect plots for the most parsimonious model (lines: mean prediction ± 95% CI) linking the dependence of the probability of long IBI *P*_long_ to (**A**) female Agreeableness *z*-score *ag*_F_ and (**B**) pair’s reproductive history defined as the sequential number *N* of the litter produced by the pair. Non-focal variable fixed at the first litter (**A**) or at the mean *ag*_F_ value in the dataset (**B**); points = individual observations coloured by *ag*_F_ value.

The probability *P*_long_ of a long IBI decreased significantly with higher female Agreeableness *ag*_F_ and with each subsequent litter *N* (*n* = 214; binomial GLMM; Fig. 1, Table 3). This pattern was much less ambiguous than for the IBIs as models relating probability *P*_long_ of a long IBI to other personality traits were clearly inferior. The top model set contained only four other models (Table S1) and essentially the same effects of Agreeableness were retained in the averaged model (Table S2).

### Litter size and infant survival

All competing models except one were plausible for litter size *B* and infant survival *s* (Table S1), suggesting only a minor influence of parent personality traits on these two components of reproductive success. The most parsimonious as well as the averaged model suggested that litter size *B* significantly decreased with higher male Conscientiousness *co*_M_ and increased with each subsequent litter *N* (*n* = 214; CLMM; Fig. S2, Table 3 and Table S2). Infant survival probability *s* decreased strongly with litter size *B* and tended to increase with the similarity index of Agreeableness |*∆ag*| (i.e. higher *s* in partners with dissimilar scores), but did not significantly vary with supplementary feeding *F* or the pair’s reproductive history *N* (*n* = 526; binomial GLMM; Fig. S3, Table 3).

In sum, the effects of personality traits on lengths of short *L*_IBI(short)_ and long *L*_IBI(long)_ IBIs, litter size *B*, and infant survival probability *s* were relatively minor (Figs. S1–S3). The respective top model set contained all or the majority of the 36 candidate models (Table S1), and the effect of most personality traits on *L*_IBI_, *B* and *s* was not significantly different from zero in the averaged models (Table S2). This suggests that the effect of personality traits on these individual fitness components is ambiguous, which contrasts with the observed strong impact of pair’s reproductive history on litter size, probability of long IBIs, and the lengths of short IBIs in the study population (Fig. 1 and Figs. S1–S2, Table 3 and Tables S1–S2).

### Fecundity rates

**Figure 2:**
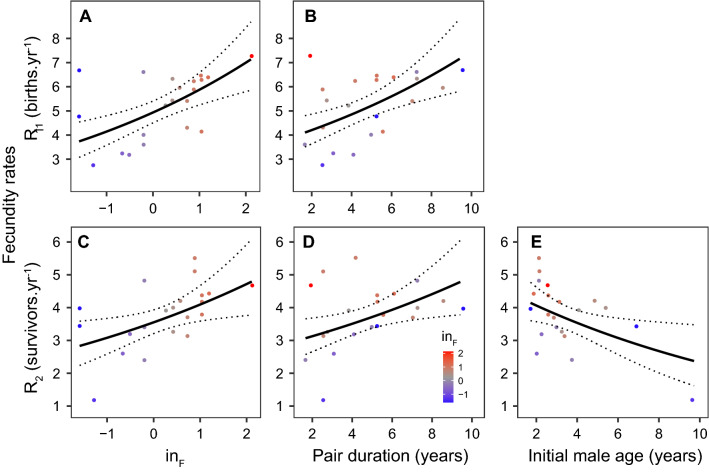
Effect plots for the most parsimonious model (lines: mean prediction ± 95% CI) linking the fecundity rates *R*_1_ (number of all offspring born per year) (**A**, **B**) and *R*_2_ (number of infants surviving until 3 months per year) (**C**, **D**) to female Inquisitiveness *z*-score *in*_F_ (**A**, **C**), pair duration *D* (**B**, **D**) and male age at pair formation (**E**). Non-focal variable fixed at the mean pair duration (**A**, **C**, **E**), mean *in*_F_ value (**B**, **D**, **E**) and mean male age at pair formation (**A**–**D**) in the dataset; points = individual observations coloured by *in*_F_ value.

The lack of clear patterns linking personality traits to most fitness components contrasted with a strong effect of Inquisitiveness on both total number of born infants (*R*_1_) and total number of successfully weaned offspring (*R*_2_) per year. The most parsimonious models revealed that both rates increased significantly with the pair duration *D* and higher female Inquisitiveness *in*_F_, and the patterns were quantitatively very similar for *R*_1_ and *R*_2_ (*n* = 21; Gamma GLM; Table 4). Moreover, *R*_2_ model residuals declined with increasing male age at pair formation, and the modified most parsimonious model including the male age showed that pairs with initially older males weaned significantly fewer offspring per year (Fig. 2, Table 4). Pairs included in our study raised on average ca. 4 infants to the age of 3 months per year (Fig. 2), well within the range of standard survival values reported in captive common marmosets^32^.

**Table 4:** Summary of the most parsimonious model for two measures of fecundity rate.

Predictors	Fecundity rate (*R*_1_)	Fecundity rate (*R*_2_)	Fecundity rate (*R*_2_)
	Estimate	Estimate	Estimate
(intercept)	1.25 (1.04–1.46)	0.94 (0.69–1.19)	1.23 (0.89–1.19)
*in*_F_	**0.17** **(0.09–0.26)**	**0.20** **(0.10–0.30)**	**0.14** **(0.03–0.25)**
*age*_M_	NA	NA	**− 0.07** **(− 0.13 to − 0.01)**
Pair duration *D*	**0.07** **(0.03–0.11)**	**0.07** **(0.02–0.11)**	**0.06** **(0.01–0.10)**
Observations (*n*)	21	21	21
df	18	18	17
Nagelkerke *R*^2^	0.548	0.482	0.619

Parameter estimates given as mean with 95% CI in the parentheses. Parameters significantly different from zero (95% CI does not overlap zero) highlighted in bold. Degrees of freedom (df) for the LMMs approximated by the Kenward–Roger method. *in* = Inquisitiveness, *age* = initial age at pair formation, _F_ = female, _M_ = male, NA = parameter not included as explanatory for the given response.

Models including other personality traits were clearly inferior. The top model set contained only two other models including Inquisitiveness (Table S1), and its effect on the fecundity rates had the same sign and magnitude in the averaged models (Table S2). Collinear personality traits could have led to our disparate results linking traits to individual components of reproductive success and fecundity rates if Inquisitiveness served as a ‘surrogate’ variable for trait combinations excluded from the candidate models (see “Materials and methods”). However, additional more complex models of fecundity rates using all linear combinations of traits included in the most parsimonious models of reproductive success components received only marginal support in the modified model selection (details not shown). This confirms Inquisitiveness as a key trait determining long-term fecundity rates in captive common marmosets.

## Discussion

This is the first study to confirm that personality traits of both partners, together with characteristics of the pair and litter, affect multiple components of reproductive success in captive common marmosets. Our analyses identified markedly variable links between personality traits and the components of reproductive success and lifetime fecundity rates. Contrary to our prediction, we found no strong evidence for the effect of parental trait matching on reproductive performance in our data but rather a direct and distinct effect of personality traits of either female or male on their reproductive success.

We identified female Agreeableness as a key personality trait driver of reproductive speed in common marmosets. More agreeable females were less likely to have long IBIs and also had shorter regular ‘short’ IBIs, indicating that these females usually conceive at the first opportunity. Agreeableness or Sociability is rarely studied in the context of animal reproductive success^24^ but, interestingly, studies in humans found a positive correlation with the number of children^4,47^. Qualities such as friendly, permissive, equable, affectionate, and predictable, may play an important role in maintaining affiliative relationships with the partner and other group members and consequently facilitate male access to mating. Pair bond quality, characterised by increased intensity of affiliative social behaviour, has been associated with IBI duration in marmosets^48^. Alternatively, the link between Agreeableness and reproduction speed might be explained by neuroendocrine mechanisms, specifically oxytocin levels. More agreeable females might have higher oxytocin levels, which regulates not only the social but also sexual bond^49^. Our results thus indicate that female Agreeableness might underpin pair bond quality and drive mating behaviour in captive marmosets.

Our results show that males with higher scores on Conscientiousness (less aggressive and assertive and more thoughtful) are more likely to sire twins compared to low-scoring males, who are more likely to sire triplets after controlling for the pair’s reproductive history. While larger litters may imply higher reproductive success, marmosets are adapted to give birth to twins and only two infants usually survive from larger litters. This trade-off between litter size and infant survival may maintain the variation in Conscientiousness in captive and possibly also in natural populations of common marmosets as in other species^50,51^, although we could not detect it in our data, possibly due to the masking effect of supplementary feeding.

So far, links between Conscientiousness and reproductive success have only been studied in humans, with a similar outcome: less conscientious fathers and mothers have more children^4,47^. Low Conscientiousness in common marmosets is characterised by items such as aggressive, assertive, or stingy and associates with bullying behaviour^40,41^. Aggressive and bold males may be more fecund (e.g. fish^3,52^, giant pandas^23^) due to more frequent mating, better sperm quantity or quality, and better physical condition^3,18^, but we could not test for these causal relationships directly. Aggressive and dominant males may also monopolise more fertile females^53^, but this mechanism is absent in captive populations with controlled pairing and lack of direct mate choice.

Importantly, the above effects of personality traits on the different components of reproductive success were relatively minor relative to the other pair characteristics and variables. Infant survival declined sharply with litter size, as found in other studies^32,34^. IBI duration increased significantly with consecutive litters, a common pattern linked to age and deteriorating physical condition of the dam^31^. Surprisingly, the probability of having triplets was higher with consecutive litters in our data. We attribute this counter-intuitive pattern to the increased weight of less active older females^36^ or to the presence of more helpers^39^. The observed propensity of having gradually larger litters also suggests the absence of reproductive senescence in marmosets^27^, but further research is needed to fully explain these phenomena.

We found strong evidence that female Inquisitiveness drives fecundity rates, reflecting lifetime reproductive success, in captive common marmosets. More inquisitive females had more offspring and weaned more infants per year—a pattern described in other species^2,15,54^. Exploration and activity are, in general, related to the acquisition of high-quality territories and food, resulting in better physical condition^55,56^. The body condition of marmoset parents might be a key factor in increased reproductive rates^39^. Due to the simultaneous pregnancy and caregiving of multiple infants, reproduction represents a substantial energetic cost to females. To compensate for this, females reduce their infant carrying efforts steadily during first two weeks postpartum^57,58^. In marmosets, heavier females should have larger litters due to more ovulations^36^ and are likely to both better feed (i.e. produce high quality milk and feed the infants more often) and carry infants^57^, which could together weigh up to 20% of female body mass^59^. Alternatively, more inquisitive mothers are likely to provide more intensive parental care (e.g. through more frequent carrying and food sharing) or show greater interest in the infants (e.g. spend more time with them) despite the large lactation costs^57^, which could improve the physical condition and development of infants^60^. The number of weaned infants per year was further driven by male’s age at the time of pairing rather than his personality type. Younger males weaned more infants per year, as found in other studies^61^. Fathers are the primary caregivers from the third week of infants’ age onwards when the infants become increasingly heavier^58^. Older males might be in worse body or health condition and thus might have difficulties carrying infants. Body condition, therefore, might be the key element to the number of weaned infants in captive common marmosets, although the underlying factors of body condition might be sex-specific. Finally, pairs that were together for a longer time had more infants and more surviving infants per year. This may be related to the increasing number of helpers^39^ or accumulated parental experience^62^.

Inquisitiveness, surprisingly, did not feature in any of the most parsimonious models for reproductive components (IBIs, litter size, and infant survival). This is because the fecundity rate (total number of infants born per year) depends not only on litter size but also on inter-birth intervals, and each of these measures of fecundity is also affected by a different set of ‘nuisance’ variables that are not (directly) linked to personality traits but affect the respective fecundity measure. Most importantly, litter size and inter-birth intervals characterise individual litters (hence with a larger set of the ‘nuisance’ variables), while the fecundity rates integrate these litter-specific data over the whole reproductive lifespan of each pair. In other words, the relatively limited effect of male Conscientiousness on litter size and the effect of female Agreeableness on the probability of a long inter-birth interval (occurring in only 14% of cases and limited mainly to early litters) were overshadowed by the effect of female Inquisitiveness when all aspects of reproductive output over the reproductive lifespan of each pair were integrated in the two measures of reproductive rates.

We did not detect Assertiveness or Patience, a personality domain unique to common marmosets^40^, as prominent drivers of any measure of reproductive performance in our data. These results suggest that multiple, but not all, aspects of animal personality may be important for different processes affecting individual reproductive success of captive common marmosets. This study is, however, not without limitations. Koski et al.^40^ reported age differences in Inquisitiveness and Agreeableness, both important predictors of reproductive success. In our study, we were not able to measure personality across individuals’ lifespans. Hence, we could not study the age-dependent effects of personality on reproductive success. Furthermore, other variables could have affected reproductive performance but were not available in our data, such as maternal litter size, parental body and health condition, parental endocrine parameters, initial condition of infants, and the number of helpers at each litter^35,36,39,63,64^. Recent studies also indicate the importance of infant-parent personality interactions^25^, a promising area for future studies.

Due to long-term pairs and high involvement of the male in infant care, we expected to find a positive effect of assortative pairing on reproductive success in common marmosets. Our results, however, did not support this prediction. Thus, assortative mating cannot be the main or exclusive mechanism maintaining different personality types in captive common marmosets. However, to fully understand the links between animal personality and individual fitness, future studies should include other mammal species with varying levels of parental care, including cooperative breeders, cover multiple measures of reproductive success and personality traits in both sexes, and different environmental conditions. Studies of wild common marmoset populations would help to further elucidate the potential confounding effect of captive conditions and reveal evolutionary mechanisms underlying personality in common marmosets.

Cooperatively breeding species represent interesting study systems as breeding is restricted to a single pair but infant care is shared among all group members. Yet, the few studies that have investigated the link between breeder personality traits and fitness have yielded ambiguous results^25,65,66^. Several factors may diminish the importance of pair matching on reproductive success in cooperatively breeding species. First, their reproductive success is bolstered by the presence of helpers and group composition^39^, which may render the personality combination of partners less important. Future studies should examine also the potential effect of helpers’ personality on infant care, number of successfully weaned infants and infant body condition. Second, the captive conditions (lower stress, enough food) might have compensated for any pair personality mismatches. The role of parental personality matching might be more critical in the wild due to different selection pressures, such as resource availability, environmental conditions, predation pressure and pathogen transmission^1^. As shown in other studies, changes in environmental conditions can lead to higher reproductive success in different combinations of parent personalities^14^. Additionally, the reproductive success of captive individuals is augmented by direct human interventions, such as hand-rearing and supplementary feeding^60^, although the latter did not significantly improve infant survival in our study population.

Further, pairs in our study population were paired randomly by keepers irrespective of their personality. Thus, the personality pair composition in our study population does not necessarily reflect the situation in the wild. Future studies should examine the variation in pair personality composition in wild common marmosets and the mechanisms underlying it, such as mate choice, social conformity or stratification of personality types in the space or time^67^. Studies on captive fish^68^, birds^69^, and mammals^70^ with biparental care have shown that females choose partners based on their personality, and these pairs have higher reproductive success. Hence, it is reasonable to assume that mate choice by personality might also occur in common marmosets and generally in cooperative breeders. Due to the varying environmental conditions and predation pressure, mate choice in common marmosets might be more important in wild populations and might occur during territorial inter-group encounters^71^. Additionally, recent experimental studies of exploration and boldness in common marmosets have shown group differences in personality traits that were produced by the social environment^72^. Hence, future studies should also focus on the potential role of social conformity on personality matching in cooperative breeders.

In conclusion, our study provides comprehensive evidence that personality traits contribute to fitness differences in captive common marmosets (c.f. Brent et al.^19^), and is the first to link parental personality traits to reproductive performance in cooperatively breeding captive non-human primates. Moreover, personality traits are often an overlooked component in captive breeding programmes and might help explain variation in reproductive success and enhance the success of ex-situ conservation efforts to save endangered callitrichid species.

## Materials and methods

### Study animals and housing

Study animals were common marmosets (*Callithrix jacchus*) housed at the Defence Science and Technology Laboratory (Dstl), Porton Down, UK. All study subjects were born in captivity. The marmosets were housed in family groups (2–12 individuals) containing a monogamous breeding pair and their offspring. The offspring stayed in their natal group until the age of 18 months. Each group was housed in an indoor enclosure (cage size: 1.52 × 1.22 × 2.15 m, temperature: 23–24 °C, humidity: 55 ± 10%) furnished with a nestbox, several branches and logs, ropes, platforms and perches (and a veranda on top of the cage), as well as various enrichment items including toys, ladders, food devices or hanging baskets. Further enrichment, including paper parcels and cardboard boxes, were given once a week, and access to a play cage was provided on a rota basis. Marmosets were housed in three rooms, each of them containing 8–12 enclosures/family groups. Food was provided twice a day, primate pellets in the morning and mixture of fruits in the afternoon, supplemented with mealworms, eggs, peanuts, dates, malt loaf and bread on alternating days. Gum arabic was provided twice a week. Vitamin D supplement was given once a week, and forage mix scattered twice a week. Water was available ad libitum. Breeding pairs were not involved in scientific studies at the facility. More information about animal husbandry and housing can be found in^32,40^.

### Personality evaluation and variables

The personality of the study subjects was evaluated in 2013 as part of a larger study by Koski et al.^40^ investigating the personality structure of common marmosets housed in three captive colonies. The Dstl subsample included 51 individuals (25 males, 26 females; mean age ± SD at personality assessment: 5.06 ± 2.51 years). The pairs included in this study were formed opportunistically by caretakers, irrespective of their personality scores and rated after pair formation (mean ± SD: 2.86 ± 2.26 years).

To evaluate personality structure, Koski et al.^40^ employed a trait rating method. Trait rating, beside experimental and common behaviour coding, is one of the commonly used methods of personality evaluation in primates^73^ in which experienced raters score the individuals, based on cumulative knowledge of their behaviour, on a set of predefined adjectives accompanied by a short description in a questionnaire. Raters assess the degree (from minimum to maximum) to which the individuals express the trait^43^. Koski and colleagues used a questionnaire with 59 items and a 7-point Likert scale (for details of questionnaire construction and description of items, see^40^). The Dstl marmosets were rated altogether by six well-acquainted raters (two raters per individual; a researcher and keepers working in the colony) with a minimum of 1-year familiarity of the subjects. Only the items with inter-rater reliability > 0 (57 out of 59 items) entered the statistical analyses (see Table S2 in the online supplementary material of Koski et al.^40^). Finally, Principal Component Analysis (PCA) was used to obtain the personality structure (for details on statistical analyses, see ^40^). The resulting personality dimensions included Agreeableness (abbreviated as *ag*), Assertiveness (*as*), Conscientiousness (*co*), Inquisitiveness (*in*), and Patience (*pa*) (for item loadings, see Table 1 and ^40^). The trait value (personality score) of a given individual on each dimension was counted using unit-weighted scores^74^. Unit-weighted score is the sum of the items that loaded saliently (i.e. ≥ |0.4|) to the dimension according to PCA weighted by − 1 or + 1 in the case of negative or positive loadings, respectively. The items that did not load are weighted by 0.

For each pair, we used the male and female trait values *T*_*m*_ and *T*_*f*_ and three measures of pair personality trait matching to quantify how individual parent personality traits and trait matching affect reproductive success. The measures of trait matching included: (1) the mean trait value *T* = (*T*_*m*_ + *T*_*f*_)/2, (2) the similarity index *|∆T|* =*|T*_*m*_ − *T*_*f*_*|* reflecting the type of mating (assortative vs. disassortative)^20^, and (3) the signed similarity index *∆T* = *T*_*m*_ − *T*_*f*_ measuring the directional difference of trait dissimilarity (see *explanatory variables* in Table 2). For a summary of mean values on individual personality dimensions see Table S3.

Before analyses, we converted the trait values *T*_*m*_, *T*_*f*_ and *T* to the respective *z*-scores *t*_M_*, t*_F_ and *t* within each trait, centered *∆T* values within each trait, and divided the centered *∆T* values and raw *|∆T|* values by the standard deviation of the respective mean trait *T*, to obtain the scaled versions of similarity indices *∆t* and *|∆t|* (Figs. S4 and S5). We detected some collinearity in the whole sets of mean traits *t*_*m*_, *t*_*f*_ and *t* and signed similarity indices *∆t* (Table S4). This did not affect the analyses, but has potential repercussions for our interpretation of the results (see “Discussion”).

### Reproductive data and variables

Reproductive data including 214 reproductive events (litters) and 560 offspring were available for 42 out of the 51 rated individuals (*n* = 21 pairs) representing a period of 14 years (2004–2018). The within-pair age difference was < 3 years (age at pairing, mean ± SD: females, 3.52 ± 2.18 years; males, 3.44 ± 1.92 years, Table S3), except for two breeding pairs. Fourteen pairs were nulliparous when established, in four pairs both the female and male had bred successfully with a different partner in the past, and in three pairs one of the breeding individuals had successfully reproduced at least once before the pair formation. Five females originating from a different facility had an unknown rearing history, and we thus did not use this variable in the analyses.

Litter sizes were constrained between 1 and 5 infants, with litters of 2 and 3 accounting for nearly 90% of all litters (Table S5). Twins and singletons were reared in their natal group. As families can rarely successfully rear litters with > 2 infants^37,60^ the following procedures were applied in an attempt to reduce infant mortality: (1) approximately half of the litters with > 2 infants received supplementary feeding sessions, where all infants were temporarily removed from the group together 4–6 times a day and supplemented with food for the first 4–7 weeks of life or (2) infants with very low survival prospects (weighing less than 27 g) and not thriving were euthanised to minimise their suffering and improve the survival prospects of their siblings. The range of practices to promote normal development and infant survival used by different facilities is described by Schultz–Darken et al.^60^. Contraception was used in cases of health problems, usually towards the end of a female’s breeding life.

We chose seven response variables covering different aspects of reproductive output from the pace of reproduction to offspring survival to fecundity rate (Table 2). That is, we included five components of reproductive success (*P*_long_: probability of having a long inter-birth interval_,_ *L*_IBI(short)_ and *L*_IBI(long)_: lengths of short and long inter-birth intervals, *B*: litter size at birth, *s:* probability of survival until the age of 3 months) and two measures of the pair’s fecundity rate (*R*_1_: total fecundity per year including stillborn offspring; *R*_2_: number of weaned offspring surviving until the age of 3 months per year).

The average gestation period in common marmosets is 143–144 days, with the first ovulation occurring 10 days after parturition^27^, suggesting minimum inter-birth intervals (hereafter ‘IBI’) of ca. 150–155 days. The first IBI was defined as the number of days from the initial pair formation to the first litter. We removed one short first IBI (131 days) from the data, as the pair was likely formed when the female was already pregnant. Most of the remaining 213 inter-birth intervals were clustered between ca. 150–160 days as expected, but some were substantially longer (up to 491 days). We used a threshold of 165 days to mark the end of the main IBI cluster, and classified all shorter intervals (*n* = 183, mean ± SD: 153.3 ± 2.8 days) as short and the remaining ones (*n* = 30, mean ± SD: 239.4 ± 69.9 days) as long (Fig. S6) (see similar distribution of IBIs in Frye et al.^33^). We then analysed the lengths of short and long IBIs separately and included the probability *P*_long_ of a long IBI (i.e. *P*_long_ = 1 if *L*_IBI_ > 165 and *P*_long_ = 0 otherwise) as another response variable.

To describe the reproductive potential of a breeding pair, we analysed the litter size at birth *B* (including the stillborn offspring). To quantify the role of infant care, we chose to analyse the probability of survival *s* until the age of 3 months for live-born offspring (we obtained very similar results for the personality traits when we included stillborn offspring; details not shown), because 3 months is the critical period during which infants depend on the (allo)parental care^27^. Moreover, infant mortality is usually highest during the first month of life^27,37^.

The two measures of fecundity rate, *R*_1_ and *R*_2_, accounted for differences in pair duration. They were, respectively, calculated as the total number of infants born and successfully raised to the age of 3 months during the pair’s lifetime divided by pair duration (years), measured from the day the pair was formed until its reproduction stopped (i.e. when the pair was dissolved or put on contraception). One pair was put on contraception for ca. 4 months and then allowed to reproduce again; we ignored this break because the next litter was born ca. 100 days after the contraception ceased and was thus conceived while the female was still on contraceptives.

Based on previous studies^32,34,37^ and preliminary data exploration, we used the following additional fixed effects characterising general life-history and ‘environmental’ conditions: (1) sequential litter number *N* as a measure of a pair’s reproductive history for litter size *B* and, as log_10_(*N*) for inter-birth durations and probability of a long IBI; (2) litter size *B* for survival probability *s*; and (3) pair duration *D* (years) for fecundity rates *R*_1_ and *R*_2_. In addition, supplementary feeding *F* (used for 55 out of 127 litters with 3 or 4 offspring) was included as a binary explanatory variable for the survival probability *s*. Parent age was unknown in one female, the initial ages at pair formation were similar across most pairs (Figs. S4 and S5), and the measure of parent age collinearity with the personality traits was low in either sex (details not shown). Moreover, parent age at the time of reproductive event correlated strongly with litter number *N*. We thus did not use parent age in the analyses but confirmed that residuals of each most parsimonious model (except fecundity rate *R*_2_, see below) did not vary predictably with initial parent age.

### Statistical analyses

All analyses were implemented in R version 4.0.2^75^. We investigated whether the components of reproductive success and fecundity rates of each pair were affected by individual male or female personality trait values, mean pair trait values, and intra-pair differences in personality.

To account for repeated data measurements for each pair, we used linear mixed effect models (LMMs) and generalised mixed effect models (GLMMs) for the analyses of the five components of reproductive success. We used binomial GLMM (implemented as *glmer* function in the *lme4* package version 1.1-21^76^) with a complementary log–log link for *P*_long_ to account for the highly uneven proportion of short and long IBIs^77^, and binomial GLMM with logit link for survival probability *s*. LMMs (implemented as *lmer* function in the *lme4* package) were used for IBI durations *L*_IBI(short)_ and *L*_IBI(long)_. Cumulative link mixed models (CLMM, implemented as *clmm2* function in the *ordinal* package version 2019.4-25^78^) were used to model discrete outcomes within the limited range of litter sizes. Fecundity rates *R*_1_ and *R*_2_ were analysed using generalised linear models (GLMs, implemented as *glm* function) with a Gamma distribution and log link as the rates were always positive. Pair identity was included as a random intercept in each GLMM and CLMM analysis.

To identify how personality traits relate to reproduction, we used the same model selection approach for each response variable. We first constructed 36 candidate models covering the null model (i.e. a model with only the relevant general life-history and ‘environmental’ conditions), 10 models with only the male or female trait *z*-scores *t*_M_ or *t*_F_, and 25 models with all five possible linear combinations of the mean scaled trait value *t* and similarity indices ∆*t* or |∆*t*| for each of the five personality traits (models abbreviated as *t*_*M*_*, t*_*F*_, *t*, ∆*t*, |∆*t*|, *t* + ∆*t* and *t* + |∆*t*|, with *t* = *ag*, *as*, *co*, *in* or *pa* in Tables S1–S3) added to the null model. Note that models *t* + ∆*t* are equivalent to models including the linear combinations of male and female trait *z*-scores *t*_M_ + *t*_F_ (i.e. both model formulations provide the same fit to the data differing only in the estimated values of the personality traits as *t*_M_ ~ (*t* + ∆*t*)/2 and *t*_F_ ~ (*t* − ∆*t*)/2), and we report only the former models. We did not include models with combinations of multiple different traits as explanatory variables in the candidate model set because we lacked a priori hypotheses for most of the response variables and we decided not to test all possible trait combinations to avoid data dredging.

We then compared these models using the corrected Akaike information criterion (AICc^79^) to identify the most parsimonious model for each response variable. We report the parameter values of each most parsimonious model along with their 95% confidence interval (CI). We deem an explanatory variable to be ‘significant’ if its 95% confidence interval does not overlap zero. We also report the ‘top model’ set, i.e. the most parsimonious model and all other plausible models with ∆AICc ≤ 6 that may reasonably describe the patterns in the data, and its conditional average (implemented in the *MuMIn* package^80^) following Grueber et al.^81^ and Richards^82^]. Residuals of the most parsimonious model of fecundity rate *R*_2_ decreased with initial male age. We thus re-ran the analysis with initial male age included as an additional predictor, and report both results (Table 4). We used the *DHARMa* package version 0.2.7^83^ to validate model residuals and the *sjPlot* package version 2.8.2^84^ to summarise the most parsimonious models.

### Ethical approval

The study was approved after review by the Stirling University Psychology Ethics Committee. All husbandry and scientific procedures were performed in accordance with legal and ethical requirements in the UK and with ARRIVE guidelines^85^.

## Supplementary Information

Supplementary Information.

## Data Availability

All data generated and analysed during this study are included in the Supplementary online materials.
