# Supplementary material for: Linking personality traits and reproductive success in common marmoset (Callithrix jacchus)
Source: Sci Rep. 2022 Aug 3;12:13341. doi: 10.1038/s41598-022-16339-4 (PMC9349211; doi:10.1038/s41598-022-16339-4)
Supplement: Supplementary file 1 — Supplementary Information. [file 41598_2022_16339_MOESM1_ESM.zip › masilkova-et-al_sem/Masilkova-et-al_SEM.pdf]

Supplementary online material: Linking personality traits and reproductive success in common marmoset (*Callithrix jacchus*)

Michaela Masilkova, David Boukal, Hayley Ash, Hannah M. Buchanan-Smith, Martina Konečná

Scientific Reports, https://doi.org/10.1038/s41598-022-16339-4

Author for correspondence: Michaela Masilkova, e-mail: michaela.masilkova@gmail.com

---

Table S1. Comparison of the 36 candidate models for the five components of reproductive success and two measures of fecundity rate. Most parsimonious model for each response variable in bold; 'top model' set with ΔAICc ≤ 6 for each response variable highlighted with grey background. Baseline model = explanatory variables include only general life-history and/or 'environmental' conditions but no personality trait; see Methods in the main text for details. ΔAICc = difference in the corrected Akaike information criterion from the most parsimonious model; df = degrees of freedom; w(AICc) = Akaike weight; rank = model rank based on ΔAICc.

[Table provided as separate Excel file]

Table S2. Conditional average of the 'top model' set for each of the five components of reproductive success and two measures of fecundity rate. For personality trait abbreviations see the main text. β = mean parameter estimate; SE = standard error of the estimate; 2.5% CI and 97.5% CI = lower and upper bounds of the 95% CI of the parameter estimate. Parameters significantly different from zero ($P < 0.05$) highlighted in bold. Dash (-) = parameter not retained in the 'top model' set; NA = parameter not included as explanatory for the given response.

[Table provided as separate Excel file]

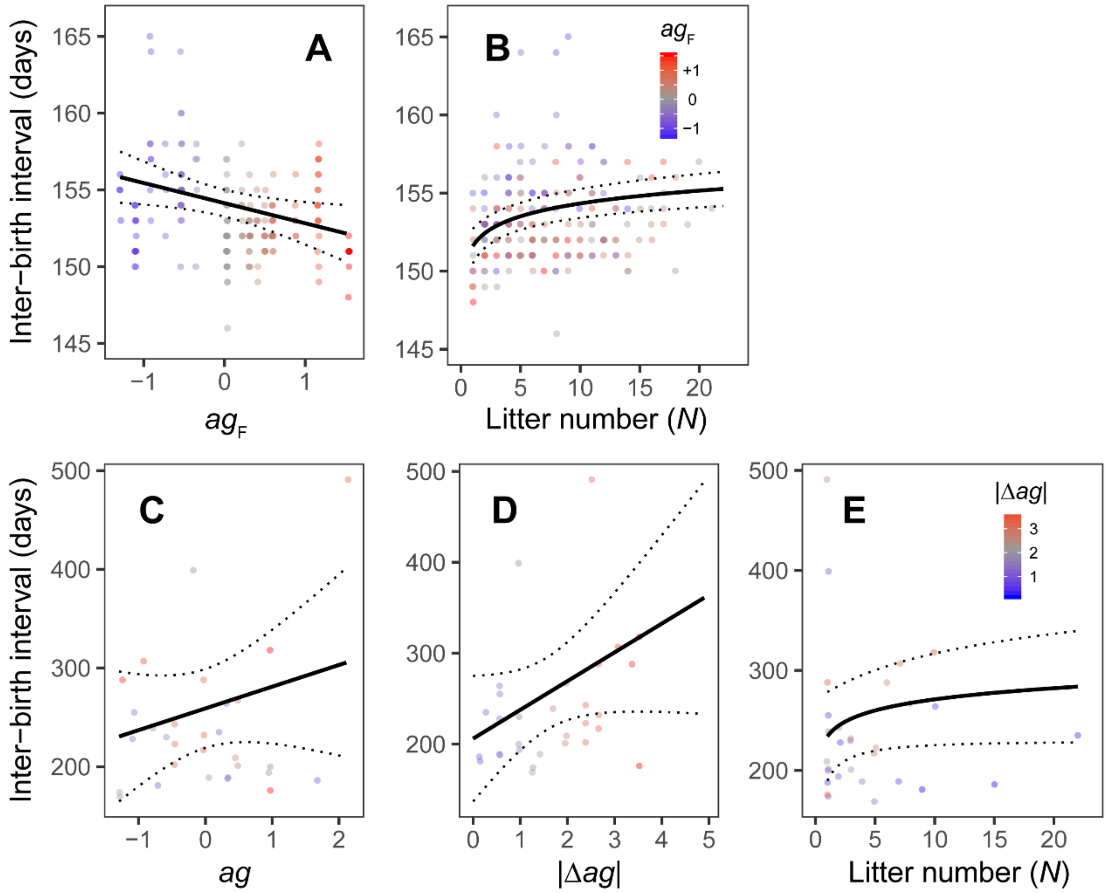

Fig. S1: Effect plots for the most parsimonious models (thick solid line and dotted lines: mean prediction ± 95% CI) linking the length of short (A, B) and long (C-E) inter-birth intervals L_{IBI} (in days) to (A) *z*-score of female Agreeableness ag_F, (C) mean pair Agreeableness *ag*, (D) similarity index of Agreeableness $|\Delta ag|$, and (B,E) pair's reproductive history defined as the sequential number *N* of the litter produced by the pair. Non-focal variables fixed at the mean value in the dataset; points = individual observations coloured by the value of ag_F (A, B) or $|\Delta ag|$ (C-E).

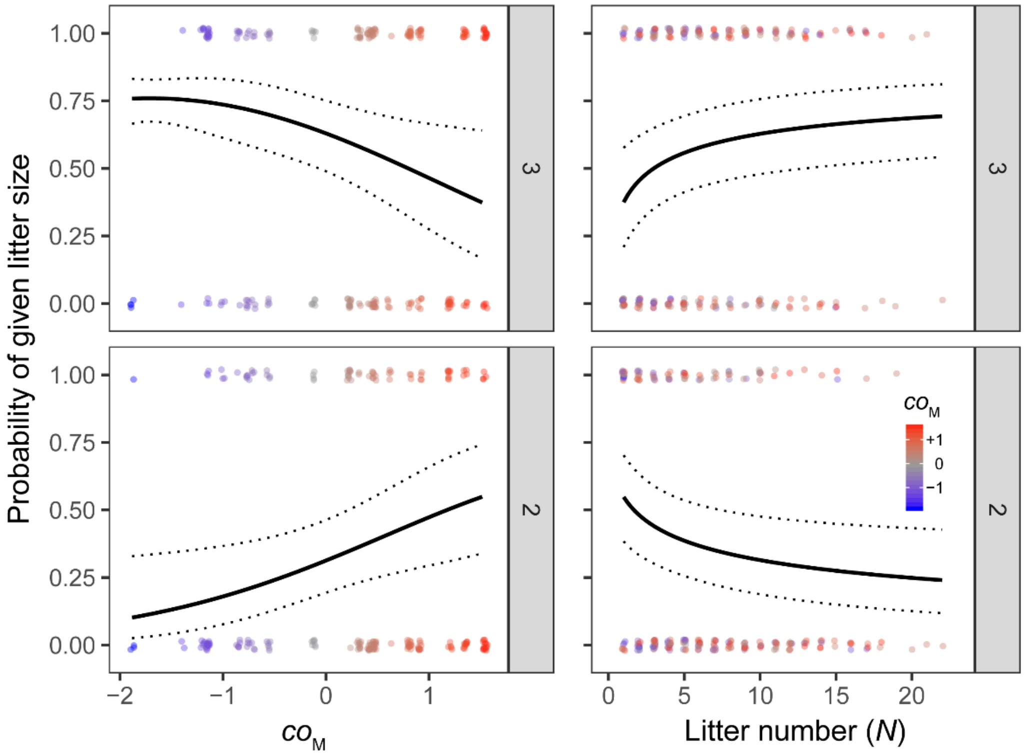

Fig. S2: Effect plots for the most parsimonious model (thick solid line and dotted lines: mean prediction ± 95% CI) linking the probability of litter sizes 2 (bottom row) and 3 (upper row) to the *z*-score of male Conscientiousness co_M (left column) and pair's reproductive history defined as the sequential number N of the litter produced by the pair (right column). Non-focal variable fixed at the mean value in the dataset; points = individual observations coloured by co_M value. Litter sizes 1, 4 and 5 with very few data omitted for clarity.

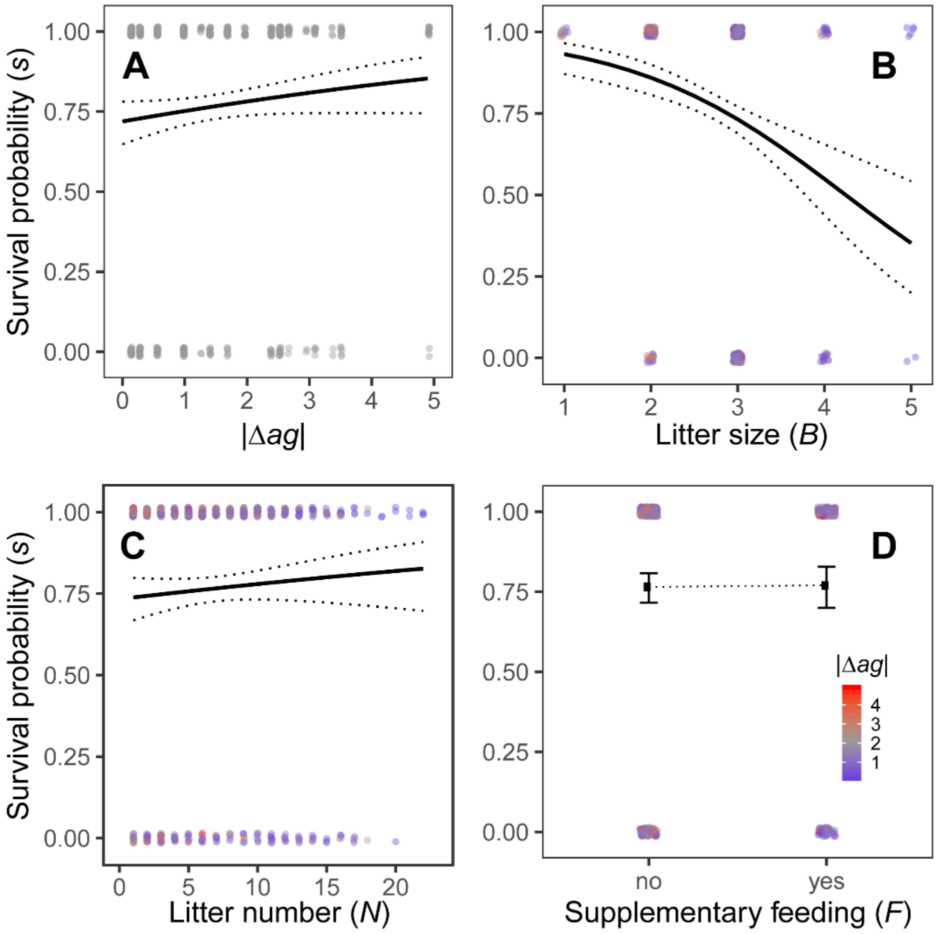

Fig. S3: Effect plots for the most parsimonious models (thick solid line and dotted lines: mean prediction ± 95% CI) linking the live-born offspring survival probability *s* to (A) similarity index of Agreeableness |Δ*ag*|, (B) litter size *B*, (C) pair's reproductive history defined as the sequential number *N* of the litter produced by the pair, and (D) supplementary feeding of the given litter. Non-focal variables fixed at the mean value in the dataset; points = individual observations coloured by |Δ*ag*| value.

Table S3. Summary of individual personality traits and age at pair formation in the 21 pairs available for the study (age in one female was unknown). T_f = female trait value; T_m = male trait value; T = average trait value of each pair; ΔT = difference in the trait values within pair (male – female); sd = standard deviation; Age (years) = in the time of personality assessment.

Trait T	mean ± sd (T_f)	mean ± sd (T_m)	mean ± sd (T)	mean ± sd (ΔT)	mean ± sd ($\lvert\Delta T\rvert$)
Agreeableness (*ag*)	49.36 ± 4.32	49.33 ± 6.25	49.3 ± 3.56	-0.02 ± 8.04	6.45 ± 4.58
Assertiveness (*as*)	-5.02 ± 5.76	-9.40 ± 6.63	-7.21 ± 5.07	-4.38 ± 7.17	7.14 ± 4.24
Conscientiousness (*co*)	-43.52 ± 10.23	-44.05 ± 10.43	-43.8 ± 7.48	-0.52 ± 14.25	11.81 ± 7.55
Inquisitiveness (*in*)	18.43 ± 3.14	16.86 ± 3.21	17.6 ± 2.49	-1.57 ± 3.94	3.19 ± 2.73
Patience (*pa*)	13.70 ± 3.94	11.36 ± 3.88	12.5 ± 3.10	-2.33 ± 4.76	3.90 ± 3.52
Age (years)	3.52 ± 2.18	3.44 ± 1.92	3.50 ± 1.86	-0.04 ± 1.80	0.90 ± 1.54

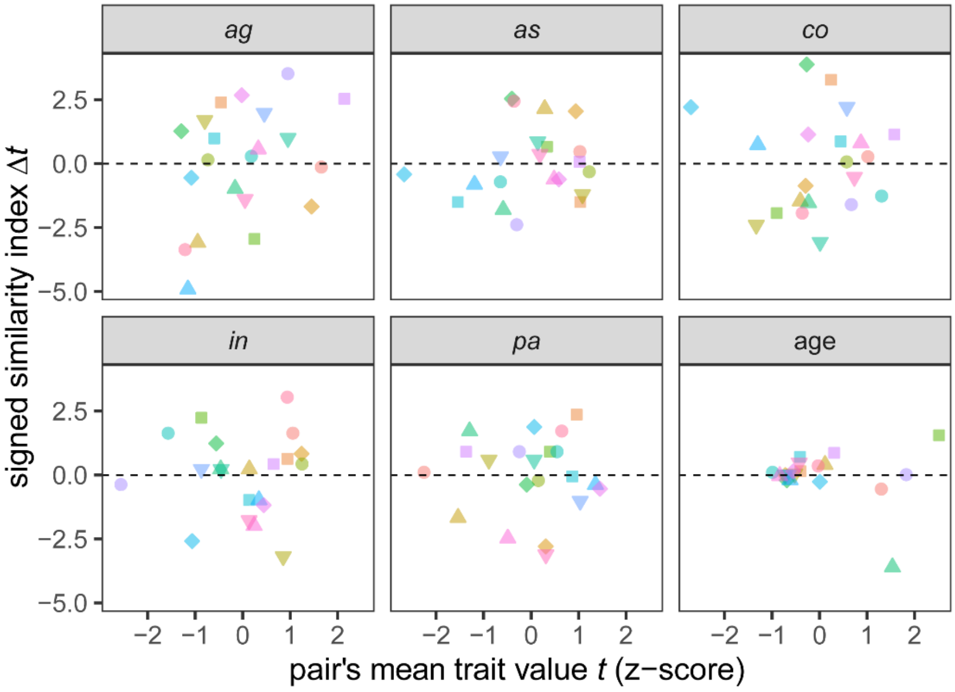

Fig. S4: Relationships between standardized mean trait value t and signed similarity index Δt within pairs. Different symbols denote different pairs. Trait abbreviations as in Table S4, age = age at pair formation. See Methods for details on data scaling procedure

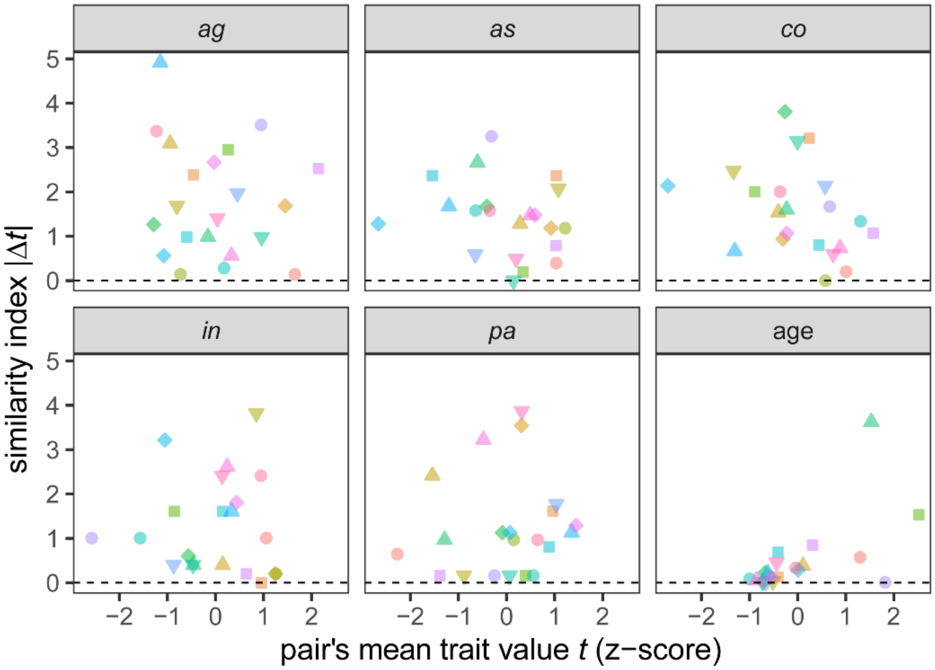

Fig. S5: Relationships between standardized mean trait value *t* and similarity index |Δ*t*| within pairs. Pair identity and trait abbreviations as in Fig. S4.

Table S4. Variance inflation factors (VIFs) of female trait *z*-scores t_F, male trait *z*-scores t_M, scaled mean trait values *t*, signed similarity indices Δ*t*, and similarity indices |Δ*t*|. Trait abbreviations: *ag* = Agreeableness, *as* = Assertiveness, *co* = Conscientiousness, *in* = Inquisitiveness, *pa* = Patience, '-' = trait not included in the given trait set. VIFs were calculated using the *corvif* function from (Zuur et al., 2010).

Trait set	ag_F	as_F	co_F	in_F	pa_F	ag_M	as_M	co_M	in_M	pa_M
1	1.31	1.93	1.98	1.52	1.10	-	-	-	-	-
2	-	-	-	-	-	1.73	3.41	1.55	2.99	1.50
3	-	-	-	-	-	1.48	-	1.44	1.12	1.04
4	2.85	3.07	2.75	3.93	2.99	3.36	7.21	3.79	9.21	5.97
5	1.63	2.76	2.30	2.17	1.42	2.12	1.53	1.59	-	2.04

Trait set	*ag*	*as*	*co*	*in*	*pa*	Δ*ag*	Δ*as*	Δ*co*	Δ*in*	Δ*pa*	|Δ*ag*|	|Δ*as*|	|Δ*co*|	|Δ*in*|	|Δ*pa*|
6	2.05	4.08	1.77	3.06	1.67	5.10	4.55	1.87	8.12	5.70	-	-	-	-	-
7	1.92	3.13	1.89	2.34	1.26	2.49	1.49	1.72	-	1.33	-	-	-	-	-
8	1.90	3.19	2.15	2.51	1.26	2.63	1.78	1.71	1.89	-	-	-	-	-	-
9	2.36	2.77	2.55	2.34	1.40	-	-	-	-	-	1.31	1.33	1.61	1.49	1.20

Table S5. Percentage of litter sizes at birth (N = 214 litters).

Litter size	Percentage
1	5.6
2	34.1
3	55.1
4	4.2
5	0.9

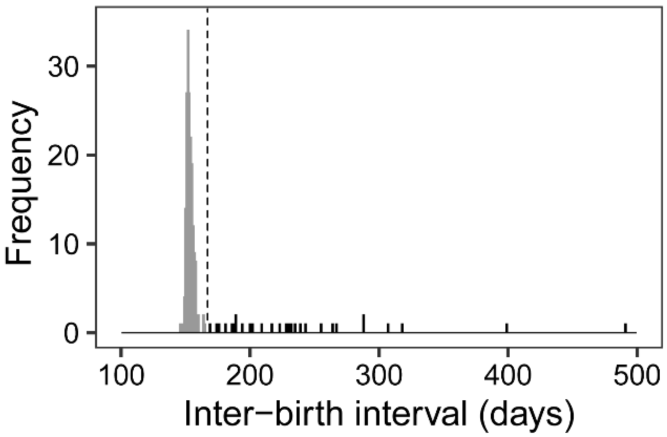

Fig. S6: Frequency of inter-birth intervals in the dataset. The dashed line separates short (grey) and long (black) inter-birth intervals.

References

Zuur, A. F., Ieno, E. N., & Elphick, C. S. (2010). A protocol for data exploration to avoid common statistical problems. *Methods in Ecology and Evolution*, *1*, 3–14. https://doi.org/10.1111/j.2041-210X.2009.00001.x
